# Politicizing science funding undermines public trust in science, academic freedom, and the unbiased generation of knowledge

**DOI:** 10.3389/frma.2024.1418065

**Published:** 2024-07-23

**Authors:** Igor R. Efimov, Jeffrey S. Flier, Robert P. George, Anna I. Krylov, Luana S. Maroja, Julia Schaletzky, Jay Tanzman, Abigail Thompson

**Affiliations:** ^1^McCormick School of Engineering, Northwestern University, Chicago, IL, United States; ^2^Harvard Medical School, Boston, MA, United States; ^3^James Madison Program in American Ideals and Institutions, Princeton University, Princeton, NJ, United States; ^4^Department of Chemistry, University of Southern California, Los Angeles, CA, United States; ^5^Department of Biology, Williams College, Williamstown, MA, United States; ^6^Department of Molecular and Cell Biology, University of California Berkeley, Berkeley, CA, United States; ^7^Independent Consultant, Pasadena, CA, United States; ^8^Department of Mathematics, University of California, Davis, Davis, CA, United States

**Keywords:** politicization of science, Critical Social Justice, STEMM funding, Meritocracy, DEI

## Abstract

This commentary documents how federal funding agencies are changing the criteria by which they distribute taxpayer money intended for scientific research. Increasingly, STEMM (Science, Technology, Engineering, Mathematics, and Medicine) funding agencies are requiring applicants for funding to include a plan to advance DEI (“Diversity, Equity, and Inclusion”) in their proposals and to dedicate a part of the research budget to its implementation. These mandates undermine the academic freedom of researchers and the unbiased generation of knowledge needed for a well-functioning democracy. Maintaining excellence in science is fundamental to the continuation of the U.S. as a global economic leader. Science provides a basis for solving important global challenges such as security, energy, climate, and health. Diverting funding from science into activities unrelated to the production of knowledge undermines science's ability to serve humankind. When funding agencies politicize science by using their power to further a particular ideological agenda, they contribute to public mistrust in science. Hijacking science funding to promote DEI is thus a threat to our society.

Do we want the mixture of students who are going to be trained to do advanced medical research to be representative of the demographic make-up of the population as a whole—or do we want whatever students, from whatever backgrounds, who have track records demonstrating a mastery of medical science that gives them the highest probability of finding cures for cancer, Alzheimer's, and other devastating diseases? *Endeavors have purposes*. Is indulging ideological visions more important than ending cancer and Alzheimer's?

–* Thomas Sowell, Social Justice Fallacies (2023)*

## 1 Introduction

Science is essential for humankind to thrive. Science is the foundation of technologies that deliver food, energy, and medicine. Scientific progress has contributed to the greatly improved human condition worldwide, including higher standards of living, lengthened lifespans, and the eradication of deadly diseases and famine. Science was the key ingredient of the industrial revolution, which propelled Western democracies to economic prosperity. Maintaining strong basic science research and education is essential for a country's security and technological competitiveness. The U.S. and other developed countries have long recognized the need for sustained support of the STEMM (Science, Technology, Engineering, Mathematics, and Medicine) fields.

The U.S. invests hundreds of billions of dollars annually in support of STEMM (AIP, (n.d.)). These funds, which ultimately derive from the taxpayers, are managed by federal funding agencies, whose role is to distribute these resources to scientists and to ensure that the funds are used effectively to produce the best outcomes for the public good. Funding agencies, therefore, play a key role in the scientific enterprise, the production of knowledge, and—ultimately—technological progress and improved quality of life.

In the U.S., the major agencies responsible for science funding are the National Science Foundation (NSF), the National Institutes of Health (NIH), the Department of Energy (DOE), the National Aeronautics and Space Administration (NASA), and the Department of Defense (DOD). Funding agencies have domains and priorities defined by their missions. For example, NSF funds fundamental research, NIH focuses on research related to human health, DOE supports research related to energy, and NASA funds research related to space. Table 1 lists the mission statements of selected agencies.

**Table 1:** Budgets and mission statements of NSF, NIH, DOE, and NASA.

**Agency**	**Annual budget (2025)**	**Mission statement**
National Science Foundation https://new.nsf.gov/about	10.2 B	NSF was established in 1950 by Congress to: •**Promote** the progress of science. •**Advance** the national health, prosperity and welfare. •**Secure** the national defense. We fulfill our mission chiefly by making grants. Our investments account for about 25% of federal support to America's colleges and universities for basic research: research driven by curiosity and discovery. We also support solutions-oriented research with the potential to produce advancements for the American people.
National Institute of Health. https://www.nih.gov/about-nih/what-we-do/mission-goals	50.1 B	NIH's mission is to seek fundamental knowledge about the nature and behavior of living systems and the application of that knowledge to enhance health, lengthen life, and reduce illness and disability.
Department of Energy. https://www.energy.gov/mission	8.6 B	**The mission of the Energy Department** is to ensure America's security and prosperity by addressing its energy, environmental and nuclear challenges through transformative science and technology solutions.
National Aeronautics and Space Administration. https://www.nasa.gov/about/	25.5 B	**Mission:** NASA explores the unknown in air and space, innovates for the benefit of humanity, and inspires the world through discovery.

Budget quoted from: “FYI: Science Policy News” (AIP, n.d.); DOE budget for DOE Office of Science only; NIH budget cited from here (request from the NIH director).

The U.S. funding agencies have excellent track records, as evidenced by the immense success that American science has enjoyed (Graham and Diamond, 1996; Urquiola, 2020). Recently, however, the function of these essential institutions has been undergoing significant changes. There has been a broad effort to use science funding to further the “Diversity, Equity, and Inclusion” (DEI) agenda (OSTP, 2022; Barabino et al., 2023; EO 13985; EO 14091). While the terms “diversity,” “equity,” and “inclusion” connote lofty goals with which the majority of Americans agree, a close look at what is actually implemented under the DEI umbrella reveals that these words represent something entirely different.

Actual DEI policies do not promote viewpoint diversity, equitable treatment of individuals based on their accomplishments, or equal opportunity for individuals regardless of their identity (e.g., race, sex, ethnicity). It can scarcely be questioned (Krylov and Tanzman, 2024) that DEI programs today are driven by an ideology, an offshoot of Critical Social Justice^1^ (CSJ) (Pluckrose, 2021; Deichmann, 2023). DEI programs elevate the collective above the individual. They group people into categories defined by immutable characteristics (race, sex, etc.) and classify each group as either “privileged” or “victimized,” as “oppressor” or “oppressed.” The goals of DEI programs are to have each group participate in proportion to their fraction of the population in every endeavor of society and to obtain proportionate outcomes from those endeavors. Disproportionate outcomes (with respect to science, such outcomes as publications, funding, citations, salaries, and awards), or disparities, are axiomatically ascribed to systemic factors, such as systemic racism and sexism, without consideration of alternative explanations (Sowell, 2019, 2023). Claims, such as “The presence of disparities is proof of systemic racism” and “Meritocracy is a myth” are propagated widely despite the vagueness of the claims and their lack of support by concrete data. Similarly, tenets that are central to DEI ideology—such as diversity is excellence, diverse teams outperform homogenous teams, and the advancement of women is impeded by biases—lack a robust evidence base, particularly when applied to science (Ceci et al., 2021, 2023; Abbot et al., 2023; Krylov and Tanzman, 2023; Ceci and Williams, 2024). Table 2 lists several examples of such axiomatic statements that funding agencies have made.

**Table 2:** Exhibits of statements made by funding agencies on the benefits of DEI.

**“The NIH BRAIN Initiative recognizes that diverse teams working together and capitalizing on innovative ideas and distinct perspectives outperform homogeneous teams. There are many benefits that flow from a diverse scientific workforce, including fostering scientific innovation, enhancing global competitiveness, contributing to robust learning environments, improving the quality of the research, advancing the likelihood that underserved populations participate in and benefit from research, and enhancing public trust.”** https://grants.nih.gov/grants/guide/notice-files/NOT-MH-21-310.html NIH: “Research shows [no references given] that diverse teams working together and capitalizing on innovative ideas and distinct perspectives outperform homogeneous teams. Scientists and trainees from diverse backgrounds and life experiences bring different perspectives, creativity, and individual enterprise to address complex scientific problems.” https://grants.nih.gov/grants/guide/notice-files/NOT-OD-24-051.html
DOE: “Transforming our understanding of nature to advance scientific discovery and U.S. energy, economic, and national security can only be accomplished by harnessing a diverse range of views, expertise, and experiences to drive scientific and technological innovation.” https://science.osti.gov/grants/Applicant-and-Awardee-Resources/PIER-Plans
NASA: “Diversity Drives Innovation. We're a diverse team united by a shared purpose. We're committed to building a workforce that reflects the diversity of the American people.” https://www.nasa.gov/careers/life-at-nasa/#Mission-and-Values

Disturbingly, CSJ is increasingly infused into every domain of the scientific enterprise—education, publishing, hiring and promotion, conferences, awards, and the allocation of funding (Abbot et al., 2023). Institutions (universities, professional associations and honor societies, and publishing houses) are subordinating scientific achievement and promise to CSJ-informed practices, such as DEI initiatives.

In this commentary, we discuss how U.S. funding agencies have begun to impose DEI requirements as a prerequisite for STEMM funding without evidence that it furthers their mission or improves the research they fund. The spread of the DEI agenda is driven both by grassroots activism and by the government, mandated by executive orders (OSTP, 2022; EO 13985; EO 14091). We note that funding agencies spend significant amounts of money on specific DEI initiatives, such as research on systemic “-isms” or specialized training and support for prioritized groups. However, in this commentary, we focus on how DEI has become a mandatory part of fundamental research proposals. The current approach to linking DEI considerations to funding decisions dilutes achievement- and merit-based criteria, which means that the funds are not necessarily used to support the best scientific ideas and projects. By requiring specific demographic outcomes, it also undermines the academic freedom of scientists to execute their research plans in an optimal manner. This diversion of public funds undermines science's ability to serve society. Moreover, when funding agencies use their power to further a particular political or ideological agenda, they contribute to public mistrust of science and scientific institutions. For these reasons, using science funding for promotion and adoption of ideologically driven DEI programs undermines the integrity of science funding, contributes to politicization of science, and represents a threat to society.

## 2 Introduction of DEI considerations into science funding decisions

Historically, U.S. funding agencies have sought to allocate funds based on the intellectual merit of the proposed ideas, the soundness of the technical plan, the feasibility of the requested budget, the alignment of the proposed research with the agency mission, and the principal investigators' (PIs) track records (Geiger, 1993; Morin, 1993; Graham and Diamond, 1996). This merit-based approach, however, is now being deemphasized. Funding agencies are introducing additional requirements not related to the proposed science, such as mandated DEI statements and plans and requirements to formulate research projects through a lens sympathetic to DEI ideology. These requirements are often vague and the related review criteria opaque. Introducing non-scientific review criteria implies a trade-off between scientific merit and other factors. At present, there is no concrete evidence that such changes in funding policies result in better scientific outcomes.^2^ We illustrate, below, the injection of DEI considerations into scientific funding decisions with examples from the DOE, NASA, NIH, and NSF.

The DOE has introduced a requirement that every research proposal include a PIER (Promoting Inclusive and Equitable Research) plan (DOE, 2023a), which “should describe the activities and strategies that investigators and research personnel will incorporate to promote diversity, equity, inclusion, and accessibility in their research projects.... The PIER Plans will be evaluated... as part of the peer review process (DOE, 2022).” The DOE webpage (DOE, 2023b) “Things to Consider When Developing a PIER Plan” “encourages” applicants to consider the composition of the project team, including project personnel and partnering institutions; the research environment; the implementation of the research project; and the scholarly and professional growth of project personnel. “This includes but is not limited to: distribution of leadership responsibilities among project key personnel; mentoring and/or training opportunities for project personnel; equitable access of project personnel to professional development opportunities; inclusive and equitable plans for recognition on publications and presentations; inclusive practices for community engagement and strategic planning meetings or events; and/or communication of research goals and results to broader audiences.” Even proposals requesting funds to support a conference need to include a PIER plan (DOE, 2023a).

**Figure 1:**
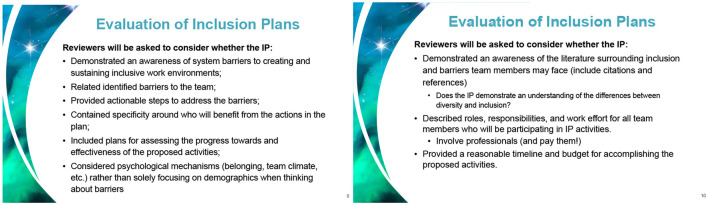
Slides from the presentation (Nahm and Watkins, 2023) describing NASA's Inclusion Plan Pilot Program (June 22, 2023).

NASA now requires research proposals to elaborate how the proposed work will “further NASA's inclusion goals.” These inclusion plans will be evaluated by panels composed *of 50% scientists and 50% DEI professionals* using the criteria shown in Figure 1, which, notably, necessitate that investigators applying for funds accept the axiomatic existence of systemic barriers preventing their research team from being inclusive. Quoting Nahm and Watkins (2023), applicants for funding are encouraged to:

- Request time or funded work effort for team members to carry out proposed IP [Inclusion Plan] activities.
- Hire IDEA [Inclusion, Diversity, Equity, and Accessibility] experts as consultants to advise the team on the proposed IP activities (*consider paying them well, too!*). [emphasis ours]
- Cite references to appropriate [i.e., papers purporting existence of systemic -isms and biases] literature in a references section separate from that of the S/T/M [i.e., technical] section.
- Request funds to support IP activities, such as training for the proposal team.

Hence, applicants must profess the belief (one not itself supported by science) that certain systemic barriers exist, dedicate time and budget for DEI activities to reduce said barriers, provide a lengthy plan (a recommendation of the Pilot Program is to extend the page limit for Inclusion Plans) and—if the funding application is successful—report on these activities.

The NIH's BRAIN initiative has implemented a requirement that, as part of the grant application, applicants submit a “Plan for Enhancing Diverse Perspectives (PEDP)” (NIH, 2021). NIH explains, however, that by “diverse perspectives,” they mean people, without regard to their scientific or scholarly perspectives. In their own words, “PEDP is a summary of strategies to advance the scientific and technical merit of the proposed project through inclusivity. Broadly, diverse perspectives refer to the *people* who do the research, the places where research is done, as well as the people who participate in the research as part of the study population.... Applicants are expected to show how enhancing diverse perspectives is supported throughout the application and how this strengthens the scientific and technical merit of the project (in terms of significance, investigator(s), innovation, approach, and environment)” (NIH, 2023a; emphasis ours).

Other NIH programs require similar DEI plans and reporting. For example, applicants are required to describe how their strategies for recruiting students and postdocs (“trainees,” in NIH's lingo) will increase the participation of underrepresented groups (NIH, 2023a,b). This requirement implicitly makes talent, skills, motivation, ability to carry out research, and scientific potential of the future trainees *secondary* to the goal of increasing diversity:

**Recruitment Plan to Enhance Diversity** **(NOT-OD-20-031)**:

The applicant must provide a recruitment plan to enhance diversity. Include outreach strategies and activities designed to recruit prospective participants from diverse backgrounds, e.g., those from groups described in the Notice of NIH's Interest in Diversity. Describe the specific efforts to be undertaken by the program and how the proposed plan reflects past experiences in recruiting individuals from underrepresented groups.

New applications must include a description of plans to enhance recruitment, including the strategies that will be used to enhance the recruitment of trainees from nationally underrepresented backgrounds and may wish to include data in support of past accomplishments.

Renewal applications must include a detailed account of experiences in recruiting individuals from underrepresented groups during the previous funding period, including successful and unsuccessful recruitment strategies. Information should be included on how the proposed plan reflects the program's past experiences in recruiting individuals from underrepresented groups.

For those individuals who participated in the research education program, the report should include information about the duration of education and aggregate information on the number of individuals who finished the program in good standing. Additional information on the required Recruitment Plan to Enhance Diversity is available at Frequently Asked Questions: Recruitment Plan to Enhance Diversity (Diversity FAQs).

*Applications lacking a diversity recruitment plan will not be reviewed. [Emphasis ours.]*

These requirements to incorporate DEI into each research proposal are alarming. They constitute compelled speech, they undermine the academic freedom of researchers, they dilute merit-based criteria for funding, they incentivize illegal discriminatory hiring practices, they erode public trust in science, and they contribute to administrative overload. “Diversity,” which is sometimes described as “diverse backgrounds” or “diverse views,” actually refers to select underrepresented identity groups (Honeycutt, 2022; Brint, 2023; Brint and Frey, 2023).

### 2.1 The integration of DEI into fundamental, unrelated research is compelled speech

**Figure 2:**
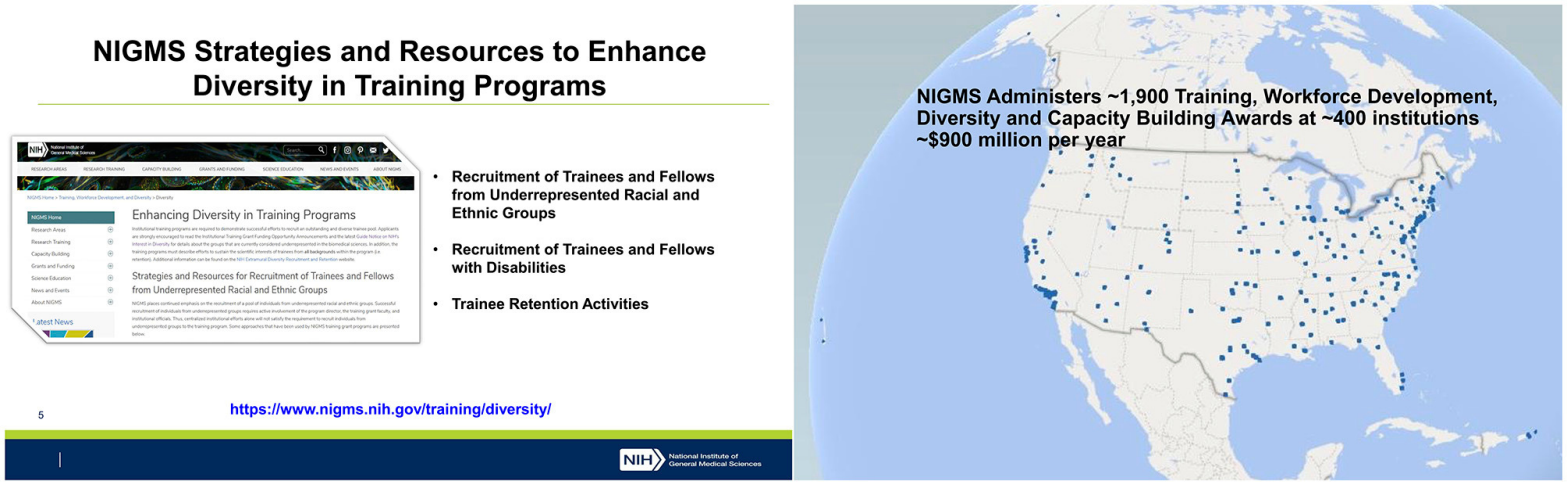
Slides from the presentation by an NIH program officer explaining DEI requirements for NIH training grants (Nelson, 2022).

DEI statements are compelled speech (AFA, 2022; Brint, 2023; Brint and Frey, 2023; Kennedy, 2024). Paraphrasing Sowell (2023): Spiritual leaders encourage—funding agencies compel. As amply documented in the context of hiring (UC Berkeley, (n.d.); Abbot et al., 2023; Brint, 2023; Brint and Frey, 2023; Sailer, 2023a,b), it is not sufficient for the applicant to make a thoughtful, reasonable statement about non-discrimination in line with applicable law; rather, the statement must fully align with DEI ideology. For example, applicants' DEI statements professing a doctrine of colorblindness have been systematically given the lowest score (UC Berkeley, (n.d.); Sailer, 2024). Similar rubrics are used by universities participating in the NIH program Faculty Institutional Recruitment for Sustainable Transformation (FIRST),^3^ which provides support to new faculty hires (Sailer, 2024). The guidelines these agencies provide to PIs (see, for example, Figures 1, 2; Nelson, 2022; NIH, 2022; DOE, 2023b; Nahm and Watkins, 2023) and funded proposals shared with us suggest that DEI plans must fully align with DEI ideology in order for the proposal to have any hope of success. For example, according to NASA's guidelines (Nahm and Watkins, 2023):

The assessment of the Inclusion Plan will be based on […] the extent to which the Inclusion Plan demonstrated *awareness of systemic barriers* to creating inclusive working environments that are *specific to the proposal team*. [Emphasis ours.]

However, not all observed disparities are due to systemic discrimination or biases (Sowell, 2019; Ceci et al., 2023; Ceci and Williams, 2024). If an applicant's institution has already overcome any systemic barriers it may have had (or the applicant believes it has done so), then the applicant must lie or the proposal is doomed to fail. The requirement to write an “inclusion plan” implies that remedial action is needed. But if access to an applicant's research team is already fair and non-discriminatory, why should an applicant be required to write an inclusion plan, a plan that requires “awareness of systemic barriers specific to the proposal team” (Nahm and Watkins, 2023), in order to succeed?

The demand to provide an inclusion plan without evidence that there is a need for one is compelled speech and an intrusion of ideology into the conduct of science. Forcing scientists to “acknowledge” and “show awareness of” systemic racism and “barriers to participation” in their institutions and teams (Nahm and Watkins, 2023), even if none can be documented, misrepresents reality, is an offense to scientists who have worked hard to establish fair and transparent hiring practices in their institutions, and is inconsistent with scientific professional ethics and, indeed, the very vocation of the scientist.

Based on feedback the authors have received from federal agencies, uncritical adoption of the doctrine of systemic racism is required, even if entirely unrelated, or even detrimental to, the proposed project. Similar to what has been observed in faculty hiring (UC Berkeley, (n.d.); AFA, 2022; Abbot et al., 2023; Brint, 2023; Brint and Frey, 2023; Sailer, 2023a,b), DEI statements informed by a doctrine of colorblindness and equal opportunity are generally rejected as “insufficient.” Proposing educational initiatives aimed at filling gaps in underrepresented candidates' skill sets is considered “deficit-centered” and is not well-received by review panels. In contrast to the well-established requirement that PIs document a track record of successful mentoring, DEI statements must contain lengthy narratives using prescribed terminology to explain how researchers and their institutions plan to uplift underrepresented groups. This conformity to ideological language is evident in funded proposals for which abstracts are in the public record (see, for example, Bamman, (n.d.); Simon, (n.d.)^4^). Scientists cannot propose plans to help overcome what they observe to be the *real* barriers to success in their field; rather, they are compelled to conform to the DEI narrative.

### 2.2 DEI vs. merit: participation vs. substantive outcome; equity vs. equality

The interaction of DEI ideology with merit raises serious concerns (Abbot et al., 2023). Introducing DEI plans into the evaluation of scientific proposals dilutes the criterion of intellectual merit, creating fertile ground for social engineering and corruption. Which proposal should be given priority for funding by DOE—the one demonstrating genuine promise in advancing solar energy research or the one promising to involve more female students? Should NIH fund the best ideas in cancer research or the best plans for achieving higher representation of LGBTQ+ researchers? We know from the history of totalitarian regimes that subjugated science to ideology, that when merit is diluted by other criteria, the chances that the most-meritorious research is funded are diminished (Graham, 1987; Josephson, 2005).

While the goal of achieving equal opportunity is uncontroversial in the scientific community and in American society at large (Gramlich, 2023), equality of outcome—so-called “equity”—is not. In the human rights literature, the “right to science” has been interpreted as a right to benefit fairly from the outcomes of science (AAAS, (n.d.)). Ensuring that the benefits of scientific progress are available to all can and should be ensured by policymakers. However, participating in the *process* of science must be merit-based, as in any field requiring specialized skill. The focus on “participation” in science treats science as an entitlement, requiring equal participation for all groups. Previously, the paradigm for allocating funding in science was to treat science as an investment and to strive to do the best possible science for the money, focusing on scientific outcomes rather than on group participation or representation. The merit-based system has historically outperformed the equity-based system in science by a wide margin (Graham, 1987; Josephson, 2005).

### 2.3 Legal considerations/civil rights laws

The interaction of DEI with the legal system is troubling. First, the demands that PIs “acknowledge” systemic racism and “barriers to participation” in their institutions (Nahm and Watkins, 2023), and insert land acknowledgments in their scientific publications [NSF, (n.d.(b))] raise grave legal concerns. The First Amendment of the Constitution of the United States strictly forbids compelling people to say things they do not believe are true. The circumstances under which government may condition grants or benefits on attesting that one holds a certain belief (e.g., “acknowledges” the truth to be this or that with respect to a contested matter), though somewhat obscure, are certainly limited (Supreme Court, 2013). At a minimum, government's engaging in such conditioning on contested questions raises significant civil liberties concerns and is in tension with core First Amendment values.

Second, there are strict laws against discrimination on the basis of race and gender, both at federal and state levels. Thus, invoking DEI explicitly attempts to circumvent existing laws. Any actual “barriers” or “systemic discrimination” can be prosecuted under existing anti-discrimination statutes, following due process.

Third, even more worrying is that successful applications require principal investigators and their home institutions to engage in practices that are likely illegal.^5^ For example, DEI “equity”-based plans for equal gender or racial participation can, in practice, only be implemented by gender- and race-preferential hiring. This is strictly illegal under civil rights employment law (EEOC, (n.d.); Title VI; Title IX).

Direct evidence of the intent of funding agencies to consider race as a factor in funding was revealed in an NIH initiative from 2021. The NIH put out a notice encouraging black scientists and those from other underrepresented groups to fill out a box for race on the funding application, which would flag their application for further consideration “even if the quality score that peer-review panels award the proposal falls outside the cutoff for most grants” (Kaiser, 2021a). The initiative has since been rescinded (Kaiser, 2021b), but NIH continues to emphasize that “diversity of the teams” is an asset in funding decisions. This creates a moral dilemma for scientists of “diverse” ancestry, as explained by Professor Kevin Williams of Temple University:

Do I deserve to jump the line? If I say yes, I may play a leading role in ending the scourge of atherosclerosis—also known as hardening of the arteries. If I play fair, I may lose the opportunity to save people around the world from heart attacks and strokes. I'm angry at the National Institutes of Health for putting me in this position. I'm even angrier it has done so in the name of racial equity....

If I refuse to identify myself as African-American, our application is more likely to lose on “diversity” grounds. It's a double wrong. Not only is the system rigged based on nonscientific—and possibly illegal—criteria; it encourages me to join in the rigging.

Truth be told, I made my decision years ago. When my study team files our application, it won't note my West African origins. If we don't get the grant, so be it. I refuse to engage in a moral wrong in pursuit of a moral good—even one as important as saving lives from the leading killer on earth. My father, who struggled against racism to achieve so much on the merits of his own work, would never forgive me for “checking the box” to grab a race-based advantage.

And no matter what happens, I can never forgive the National Institutes of Health for reinjecting racism into medical research (Williams, 2024).

Funding agencies attempt to circumvent the laws prohibiting them from basing funding decisions on race or ethnicity by cloaking DEI requirements in nebulous language (NIH, 2019; Renoe, 2023) and by disguising racial preferences and even quotas as “diversity of backgrounds” and unequal treatment as “broadening participation of underrepresented groups.” The determination of which groups to treat as underrepresented and worthy of special treatment is highly subjective, as Americans hold many identities and can be split up in a multitude of ways. In practice, implementing equity-focused DEI programs means preferring members of some groups over others (Kendi, 2019). To paraphrase Orwell, all groups are equal, but some groups are more equal than others (Orwell, 1945).

The evaluations of submitted DEI plans are not open to public scrutiny. Agencies run diversity-focused programs but refuse to give guidance on how to determine eligibility for them; they are careful to state that compliance with all applicable employment laws is the responsibility of the host institution. However, DEI metrics, which must be reported annually to the funding agency, are criteria for renewal (NIH, 2023b). It remains unclear how a principal investigator is supposed to be non-discriminatory in hiring and at the same time fulfill *de facto* DEI quotas for renewal. In this way, programs are developed that are *de jure* “open to everyone,” but *de facto* allocated according to identity metrics, reminiscent of the pre-civil rights era in the U.S.

**Figure 3:**
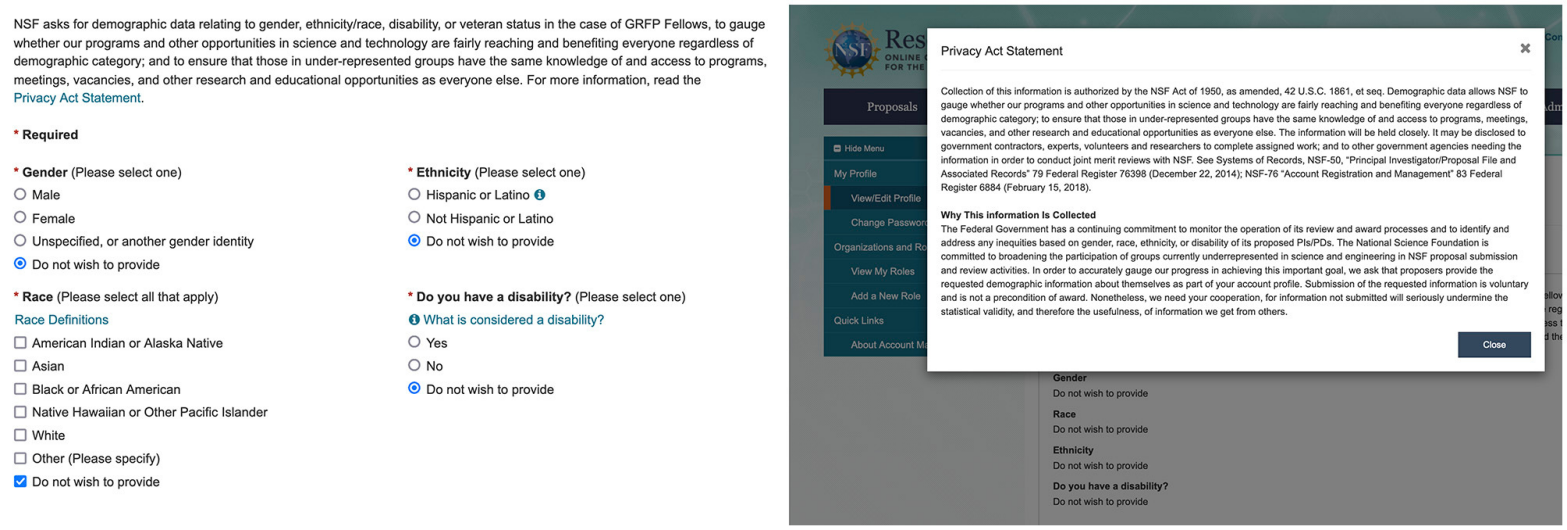
Screenshot of grants.gov website used to submit proposals to NSF and to manage reporting of the funded grants. The PIs are required to complete their demographic profile before they are allowed to use the website.

The extensive collection of demographic information by the funding agencies is also concerning. For example, the portal for managing NSF grants (grants.gov) requests users (prospective and funded PIs) to report their demographic details (see Figure 3). The stated purpose of this request is “to gauge whether our programs and other opportunities in science and technology are fairly reaching and benefiting everyone regardless of demographic category; and to ensure that those in under-represented groups have the same knowledge of and access to programs, meetings, vacancies, and other research and educational opportunities as everyone else.” However, the cited Privacy Act statement (Plimpton, 2020) does not indicate that the collected information will be used only in aggregated form and that demographic data of the PIs will not be visible to the program officers. On the contrary, it states, “The information on proposal forms will be used in connection with the selection of qualified proposals; and project reports submitted by awardees will be used for program evaluation and reporting within the Executive Branch and to Congress. The information requested may be disclosed to qualified reviewers and staff assistants as part of the proposal review process.” (Plimpton, 2020). The NSF Privacy Act (NSF, 2018) clarifies that the purpose of the online portal system is to “provide a dashboard for administrators to easily manage NSF system roles for their organizations” and to “provide demographic data that NSF may track over time in order to review and evaluate NSF programs.”

### 2.4 Politicization of science erodes public trust

DEI politicizes science, which erodes public trust in scientific institutions, scientists, and the entire scientific enterprise.

Public trust in institutions is essential for a functioning democracy. Science funding ultimately depends on the goodwill of the voting and tax-paying public. When federal funding agencies infuse political agendas into their function, they contribute to public mistrust in the process by which science is funded. When universities become complicit by subjugating their mission of truth seeking to ideologically driven DEI programs, they contribute to public mistrust in scientific institutions (Kennedy and Tyson, 2023). Should the public withdraw its support for science, loss of funding will ultimately ensue, with attendant detrimental consequences to the nation.

The politicization of science by DEI also erodes the trust in scientists and the scientific enterprise itself (Kahan, 2010, 2015) that is required for experts, the public, and legislators to effectively work together to solve pressing problems, such as climate, energy, and pandemics. Mistrust in science also provides fertile ground for science denial, conspiracy theories, and political opportunism.

### 2.5 Administrative overload

Scientists are already overwhelmed with administrative tasks. Time spent writing a winning DEI statement is time not spent thinking about how to solve a difficult scientific problem. Mervin Kelly, Director of Bell Labs, one of the most innovative and impactful US research institutions of the 20th century, famously stated about the work environment:

We give much attention to the maintenance of an atmosphere of freedom and an environment stimulating to scholarship and scientific research interest. It is most important to limit [the scientist's] work to that of research (Georgescu, 2022).

Yet, by some estimates, top researchers today spend more than 50% of their time writing grant applications (Belluz et al., 2016), many of which comprise hundreds of pages of documents and forms required by the federal government, the majority of which are unrelated to the science being proposed. DEI statements and reporting requirements add to that. These statements and reports are difficult and time-consuming to write as the applicant is expected to use prescribed, often convoluted, DEI terminology, reflecting, for example, on how “inclusion” differs from “diversity” (Nahm and Watkins, 2023). An example of a successful DEI plan from a funded NIH proposal gives an idea of what is expected from PIs (Bamman, (n.d.)).

For some NIH training grants, multilayered diversity plans (Nelson, 2022) are required for the mentoring faculty, for the leadership team, and for the program's directors. In addition, NIH requires DEI plans for recruitment of scholars (NIH, 2022, 2023b) and mandates diversity training for mentors—“training the trainers”—and a plan for how the scholars themselves can mentor other students to propagate DEI [NIH, (n.d.(a)), 2024b; Nelson, 2022]. Even scholars who are underrepresented themselves, and sometimes have overcome significant hardship, must articulate how they plan to promote DEI, at the expense of focusing on their own research plans.

### 2.6 Why is this happening?

Why are funding agencies participating in activities that are arguably unrelated to their stated missions, and in important respects even undermining them? The DEI movement has undeniable grassroots components, comprising both sincere, activist scholars and cynical opportunists who use DEI to advance their careers. But these elements alone cannot explain why funding agencies have so radically veered from their original missions.

In fact, the mandate that funding agencies implement DEI comes directly from the White House. Executive Order 13985, titled “Advancing Racial Equity and Support for Underserved Communities Through the Federal Government,” directed all federal agencies to allocate resources to DEI and to incorporate “equity” into their decision making as a principle (EO 13985).

EO 13985 begins with acknowledging that equal opportunity is a bedrock of American democracy and that historic injustices have denied this equal opportunity to certain groups and individuals. It cites existing gaps and inequalities:

For purposes of this order: (a) The term “equity” means the consistent and systematic fair, just, and impartial treatment of all individuals, including individuals who belong to underserved communities that have been denied such treatment, such as Black, Latino, and Indigenous and Native American persons, Asian Americans and Pacific Islanders and other persons of color; members of religious minorities; lesbian, gay, bisexual, transgender, and queer (LGBTQ+) persons; persons with disabilities; persons who live in rural areas; and persons otherwise adversely affected by persistent poverty.

If “consistent and systematic fair, just, and impartial treatment of all individuals” means equality of opportunity and equitable treatment of people's accomplishments based on their merit, we're all for it. However, the Order goes on to make clear that the goal is not to achieve equal opportunity and equitable treatment, but to achieve equal outcomes for identity groups. The Order conflates racism in the past with disparities in the present and equitable treatment with equal outcomes. It attributes unequal participation in the present to alleged discrimination in the present. It charges the Domestic Policy Council with the task “[of] remov[ing] systemic barriers,” thus implicitly asserting the existence of such barriers in the present. It calls for “redress[ing] inequities,” “affirmatively advancing equity,” and “allocating Federal resources in a manner that increases investment in underserved communities, as well as individuals from those communities.” Whatever is to be said about such goals in relation to, say, social welfare programs, we question their value and appropriateness for science funding.

The American public generally agrees with equal opportunity as a goal, but not with identity group-based preferences (Gramlich, 2023). The courts have held the same, with equal opportunity mandated by civil rights law since the 1970s and identity group-based preferences in college admissions struck down by the Supreme Court in 2023. But EO 13985 turns the argument that equity/preferences is unfair on its head and instead claims that equity/preferences is a *prerequisite* for “equal opportunity”. In this way, supporting equal opportunity tacitly requires supporting race- or identity-based preferential treatment as a *precondition* to equal opportunity.

The words “merit,” “excellence,” and related words such as scientific “achievement” and “accomplishment,” are conspicuously absent in the six-page Order (EO 13985). It is clear that the EO does not call for equal recognition for equal merit (achievement, accomplishment, promise), but aims to give preferential treatment to specific identity groups (listed above) in an attempt to close achievement gaps and redress past injustices.

The theme that “bias, discrimination, and harassment plague the science and technology ecosystem, from school to workforce and beyond” is continued in the official statement “Equity and Excellence: A Vision to Transform and Enhance the U.S. STEMM Ecosystem,” issued by the Office of Science and Technology Policy (OSTP, 2022). Citing unequal outcomes in the distribution of research funding—e.g., that black PIs were funded at a lower rate than white PIs—the document calls for sustained intervention to “close the [research] funding gap and support researchers and communities who have been historically excluded from access to key resources.” The document is replete with DEI vocabulary (“equitable access and outcomes,” “holistic support,” “systemic barriers—including bias, racism, sexism, ableism, exclusion, discrimination, cultural disincentives,” “structural barriers”). Again, no mention of merit and the like.

The goal of promoting “equity” in science is reinforced in Executive Order 14091 (EO 14091). Titled “Further Advancing Racial Equity and Support for Underserved Communities Through the Federal Government,” it explains how equity is to be implemented in various domains, and specifically calls for the “promot[ion] [of] equity in science.” It lays out specific DEI requirements for federal agencies, including NASA and NSF, such as the following:

The Administrator of the National Aeronautics and Space Administration, the Director of the National Science Foundation [...] (agency heads) shall, within 30 days of the date of this order, ensure that they have in place an Agency Equity Team within their respective agencies to coordinate the implementation of equity initiatives and ensure that their respective agencies are delivering equitable outcomes for the American people.

These orders do not mandate equitable treatment of applicants based on achievement and promise, i.e., merit; rather they mandate that research funding be distributed “equitably”—i.e., proportionally to demographic representation—among identity groups.

The foregoing EOs are enforced by the Office of Management and Budget (OMB), which conducts DEI audits “in partnership” with each agency and makes recommendations to address DEI concerns. At the same time, OMB plays a role in determining both the level of funding for each agency and how funds are allocated to programs within each agency [NSF, (n.d.(a))]. OMB thus has the power to allocate science funding on the basis of DEI compliance, potentially at the expense of scientific merit.

As ordered, the funding agencies have responded to these government mandates by establishing DEI offices, rolling out DEI initiatives, instituting DEI plans, and introducing DEI reporting requirements [NIH, (n.d.(b)); NSF, (n.d.(b)), 2022; NASA, 2022; DOE, 2023a,c]. The DOE Equity Action Plan (DOE, 2023a), for example, pledges to “increase participation by individuals and institutions that are underrepresented in DOE's research and development (R&D) programs supported through financial assistance.” It proposes to “expand Tribal engagement and stakeholder engagement across DOE.” In a truly Orwellian manner, DOE promises to “update the DOE Merit Review Program to improve equitable outcomes for DOE awards” (DOE, 2023c).

The NSF Equity Plan [NSF, (n.d.(b))] includes activities and steps “addressing sexual and other forms of harassment, optimizing demographic data collection in support of equity assessments, increasing participation of disadvantaged entities [including Minority Serving Institutions (MIs)], on Federal Acquisition Regulation-based solicitation and awards, and removing barriers to enhanced participation by indigenous and Native American communities.” Among specific steps, NSF now requires researchers who use selected astronomical facilities to include land acknowledgments in their work [NSF, (n.d.(b))]. As an example of how the plan is being implemented, teams applying for Centers for Chemical Innovation grants must now include a Diversity and Inclusion Plan (recommended length 2 pages) “to ensure a diverse and inclusive center environment, including researchers at all levels, leadership groups, and advisory groups,” in addition to a mandatory broader impact section (recommended length 8 pages) that should include plans for broadening participation by underrepresented groups (NSF, 2023).

NIH's activities toward advancing racial equity [NIH, (n.d.(b))] include an invitation to “Take the Pledge,” which includes committing to an idea that “equity, diversity, and inclusion drives success,” “setting up a consultation with an EDI [DEI] liaison,” and “ordering the ‘EDI Pledge Poster' (or … creat[ing] your own) for your space and hav[ing] your team sign it” [NIH, (n.d.(c))].

### 2.7 Who or what does DEI benefit?

It is not clear how science is supposed to benefit from the imposition of DEI ideology and programs on funding decisions. To our knowledge, there have been no quantitative studies demonstrating that any DEI intervention has increased the quality or quantity of scientific output (see text footnote 2). On the other hand, one group that clearly benefits is the DEI bureaucracy itself: specialized hires within agencies^6^ and universities, and highly paid DEI experts and consultants.^7^ Not unlike lobbyists, DEI experts advise agency staff to create positions for themselves or their colleagues in winning grant proposals (Nahm and Watkins, 2023). Since DEI statement requirements are nebulous and confusing, unsurprisingly, the solution is to hire a well-paid consultant [NIH, (n.d.(c)); Nahm and Watkins, 2023]. Some agencies, such as NASA, even make the inclusion of paid professional DEI consultants in the project mandatory (Nahm and Watkins, 2023; see Figure 1— “pay them well”). These highly paid consultants often have no expertise in the conduct of science. Hiring them requires administrative effort and diverts significant funding away from science. NSF also recommends allocating 5–10% of the total budget to “broader impact” activities, which heavily emphasize DEI (Renoe, 2023). This continues an unfortunate trend of prioritizing documentation, compliance, and activities that do not contribute to actual scientific work over innovation in federally funded research. Quoting from a brief submitted to the Canadian House of Commons by 40 university professors, DEI “is self-perpetuating, has no end goal, and uses flawed metrics.” (Horsman et al., 2024).

### 2.8 DEI in perspective

Although our commentary focuses narrowly on the current U.S. funding scene, the spread of DEI ideology and its intrusion into science has been limited neither geographically nor temporally. Abbot et al. (2023) provided examples of current DEI-informed policies in Europe, United Kingdom, Canada, Australia, and New Zealand [see also a brief submitted to the Canadian House of Commons by 40 academics calling for the abolition of “costly and inequitable” DEI initiatives (Horsman et al., 2024)]. Historically, ideological control of the scientific enterprise has been practiced by totalitarian regimes, notably, by Maoist China and Soviet Russia, with disastrous consequences for science and technology in both countries (Graham, 1987; Josephson, 2005; Krylov, 2021). Identity-based social engineering was practiced to the extreme in Nazi Germany, where universities were purged of non-Aryans by government decree (Deichmann, 1999, 2023). In the USSR, professional advancement was conditioned on class (favoring the proletariat and disfavoring the educated “intelligentsia”), ethnicity (e.g., participation of Jews was limited by quotas), and ideological compliance (Graham, 1987; Alexandrov, 2002; Shifman, 2005; Gruntman, 2022; Krylov, 2022).

To illustrate the pernicious effect of the ideological control of science, consider Lysenkoism—a dark period in Soviet science when the field of genetics was denounced as a “bourgeoise pseudoscience,” scores of scientists fired and jailed, with many—including brilliant biologist Nikolai Vavilov—perishing in the Gulag (Graham, 1987; Kolchinsky, 2014; Reznik, 2017; Deichmann, 2023). The key player in this devastating attack on science was Trofim Lysenko, a poorly educated agronomist who promoted a number of scientifically flawed ideas—such as the rejection of genes and the belief in the complete malleability of phenotypes. He succeeded in destroying his scientific opponents not by beating them in a scientific debate, but by having the support by the Communist Party, including Stalin himself. Lysenko's rise to power was partially because of his pedigree—he was the poster child of a “people's scientist” because he came from a family of poor peasants. He did not learn how to read until age 14, and the Soviet press lovingly called him the “barefoot scientist.” In contrast, his main opponent, Nikolai Vavilov, was suspect because of his class (the “intelligentsia”). This was official Party policy—to rapidly promote members of the proletariat into leadership positions in agriculture, science, and industry. Lysenko also formed an alliance with a Marxist ideologue (Isaak Prezent), who cleverly used philosophical arguments, such as claiming that genetics contradicted Marxist-Leninist doctrine.

Lysenko and his supporters destroyed the entire field of genetics in the USSR, suppressing research there for more than a decade. Lysenko's bogus science was used to introduce flawed agricultural practices on a large scale, causing devastating famines in the USSR and China.

## 3 Vision/summary

Science is a national and, indeed, global public good that has afforded us an unprecedented standard of living and wellbeing, the eradication of diseases, healthier lives, and increased lifespans. Although the benefits have not been shared equally, scientific progress has benefitted all members of society, including members of marginalized groups.

Science is also an essential component of a well-functioning democracy. By providing a foundation for technological developments, science is instrumental in maintaining America's global competitiveness and its national security (Deift et al., 2021; Eaglen, 2023; Luckenbauch, 2023). Funding for science is limited—about 3% of GDP for the US. It is imperative that this funding be used efficiently and effectively to advance knowledge, produce innovation, maintain national security, improve health, deal with unforeseen crises and challenges, and find solutions to environmental and other problems.

Science and ideology are fundamentally different things. Science, by its nature questions assumptions; to flourish, it requires freedom of inquiry and the free exchange of ideas. Ideology is hostile to such freedoms. Historically, when ideology has invaded science, stagnation and collapse have ensued (Graham, 1987; Josephson, 2005; Krylov, 2021).

In order to be effective in advancing knowledge, delivering innovation, and serving humankind, science must:

- Operate free from ideology and politicization in a climate of open inquiry.
- Center the autonomy of researchers, acknowledging their expert role, allowing them to do what they do best and minimizing time spent on administrative, compliance and reporting duties, and so-called “broader impact” activities.

Systemic disparities in opportunity, especially those related to socio-economic status, are real and well-documented. Solid family structure, access to healthcare, good nutrition, an environment free from violence and drugs, high-quality preschool and K−12 education are necessary to nurture the next generation of scientists, but they are not equally available to all Americans. Rather than attempt to institute “equity” by mandating proportional participation through the manipulation of grant funding, we believe that increased efforts should be made to promote equality of opportunity as early in people's lives as possible so that young people who aspire to standing in any field, including scientific fields, can succeed on merit (Abbot et al., 2023, 2024; Loury, 2024).

DEI initiatives such as those related to grant funding have taken the place of efforts to investigate and solve the underlying issues leading to inequities—the root causes that prevent all Americans from achieving their potential. DEI is based on the fallacy that a fair and equitable society can be achieved by mandating proportional participation in a highly competitive, achievement-based activity, such as science (Sowell, 2023). Indeed, some DEI efforts have been outright harmful to the very groups they purport to uplift. For example, in the name of “equity,” public school K−12 math curricula have been systematically dismantled (Deift et al., 2021; Evers and Hofer, 2023), and this most-strongly disadvantages high-achieving students of families that cannot afford private schools (McWhorter, 2021): potential future scientists with minority backgrounds—precisely those who DEI efforts in science purportedly aim to help. Likewise, identity group preference programs have harmed minority students admitted to universities that did not match their level of preparation (Heriot and Schwarzchild, 2021; Sowell, 2023).

Observed disparities in participation in the scientific enterprise should be systematically investigated and analyzed for their root causes before concluding that they are the result of discrimination (Sowell, 2019). When discrimination is identified, it should be remedied by enforcing existing civil rights laws. Attempting to fix disparities by social engineering is ineffective, unfair, and potentially illegal. As Chief Justice Roberts stated:

The way to stop discrimination on the basis of race is to stop discriminating on the basis of race,

a view fundamentally opposite to the CSJ approach, as succinctly expressed by author Ibrahim Kendi:

The only remedy to racist discrimination is antiracist discrimination. The only remedy to past discrimination is present discrimination. The only remedy to present discrimination is future discrimination (Kendi, 2019).

We recommend that all federally funded agencies focus on their primary role in generating the maximum public good for the public funds spent, and not take on the role of promoting any ideological agenda. Funding agencies should abolish DEI requirements and focus instead on funding proposals based on their merits. Strengthening K−12 education and merit-based practices is the path toward equal opportunity, fair distribution of resources, and the best science to the benefit of all (McCloskey, 2016; Wooldridge, 2021; Abbot et al., 2023, 2024).

## Author contributions

IE: Investigation, Writing – review & editing. JF: Conceptualization, Investigation, Writing – review & editing. RG: Investigation, Writing – review & editing. AK: Conceptualization, Investigation, Writing – original draft, Writing – review & editing. LM: Investigation, Writing – review & editing. JS: Conceptualization, Investigation, Writing – original draft, Writing – review & editing. JT: Conceptualization, Investigation, Writing – original draft, Writing – review & editing. AT: Investigation, Writing – review & editing.

## Footnotes

^1^CSJ is rooted in Critical Theories and postmodernism (Lyotard, 1984; Delgado and Stefancic, 2001; Aylesworth, 2015; Celikates and Flynn, 2023; Britannica, 2024).

^2^We make this statement with some authority, as several of us are involved in a comprehensive review of the literature relevant to the claim that diverse research teams produce better science.

^3^From the program description: “The NIH Common Fund's **Faculty Institutional Recruitment for Sustainable Transformation (FIRST)** program aims to enhance and maintain cultures of inclusive excellence in the biomedical research community. ‘Inclusive excellence' refers to cultures that establish and sustain scientific environments that cultivate and benefit from a full range of talent. NIH aims to facilitate institutions in their building a self-reinforcing community of scientists, through *recruitment of a critical mass of early-career faculty who have a demonstrated commitment to inclusive excellence*. The program also seeks to have a positive impact on faculty development, retention, progression, and eventual promotion, as well as develop inclusive environments that are sustainable.” (NIH, 2024a; emphasis ours).

^4^From *NURTURE: Northwestern University Recruitment to Transform Under-Representation and achieve Equity*: “NURTURE aims to disrupt systemic barriers that impede full participation of biomedical research scientists from underrepresented groups (URG) by investing in inclusive cultural change within our institution. We acknowledge that systemic racism has persisted in biomedical science, including at Northwestern. We are committed to dismantling the structures that have allowed racism and bias to persist and impeded the scientific careers of too many URG scholars. NURTURE proposes to transform siloed fiefdom structures to transdisciplinary Scientific Neighborhoods that will foster growth and accomplishment in the research, career, and personal trajectories of URG faculty.” (Simon, (n.d.)).

^5^See, for example, statements from the abstracts of proposals funded by the NIH FIRST program that explicitly plan hiring based on minority status (NIH, 2024a): “Cornell FIRST will support the hiring and retention of 10 new assistant professors from groups underrepresented in their fields, while transforming institutional climate into a culture of inclusive excellence.... Cornell's FIRST program features interdisciplinary hiring of faculty underrepresented in their fields.” (Cornell FIRST). “The overall goal of the University of California San Diego (UCSD) NIH... FIRST... Program is to:... promote institutional excellence by hiring a diverse cohort of faculty.... The objectives are to:... conduct recruitment of new faculty, outline institutional commitments, and develop recruitment committees based on commitments to diversity, equity and inclusion.” (UCSD FIRST). “This new model will seek to hire three cohorts of underrepresented scholars, who are committed to diversity in the academy.” (University of Maryland FIRST).

^6^To “embed and integrate Diversity, Equity, Inclusion, and Accessibility (DEIA) into NSF's policies, practices, and culture,” NSF allocated two million dollars and created DEIA implementation team of 25 staffers including newly created top-level position of Chief Diversity Officer (see Appendix B in NSF, 2022).

^7^According to salary.com, the average salary of a DEI director in California is $227,773. DEI consultant hourly rates vary, with the median being $53/h (according to ZipRecruter.com). The NOVA collective, a Chicago based DEI company, gives the prices for their services on their website. Single instructor-led training sessions cost $500–10,000, E-learning modules cost $200–5,000, Keynotes go at $1,000–30,000. Consulting monthly retainers cost between $2,000 and 20,000, and single consulting deliverables cost $8,000–50,000.
